# Pesticide Mixtures in Surface Waters of Two Protected Areas in Southwestern Germany

**DOI:** 10.1007/s00128-023-03830-5

**Published:** 2023-12-12

**Authors:** Anna Schemmer, Jakob Wolfram, Alexis. P. Roodt, Sascha Bub, Lara L. Petschick, Larissa Z. Herrmann, Sebastian Stehle, Ralf Schulz

**Affiliations:** 1 Institute for Environmental Sciences, iES Landau, Rhineland-Palatinate Technical University Kaiserslautern-Landau, Fortstrasse 7, D-76829 Landau, Germany; 2 Eusserthal Ecosystem Research Station, Rhineland-Palatinate Technical University Kaiserslautern- Landau, Birkenthalstrasse 13, D-76857 Eusserthal, Germany

**Keywords:** Drinking water, Biosphere reserve, Environmental risk, Event sampling, Agriculture, Pesticide mixtures

## Abstract

Pesticides enter non-target surface waters as a result of agricultural activities and may reach water bodies in protected areas. We measured in southwestern Germany pesticide concentrations after heavy rainfalls in streams of a drinking water protection area near Hausen (Freiburg) and in the catchment of the Queich (Landau), which originates from the biosphere reserve Palatinate Forest. On average, 32 (n = 21) and 21 (n = 10) pesticides were detected per sample and event in the area of Hausen (n = 56) and in the Queich catchment (n = 17), respectively. The majority of pesticides detected in > 50% of all samples were fungicides, with fluopyram being detected throughout all samples. Aquatic invertebrates exhibited highest risks with 16.1% of samples exceeding mixture toxicity thresholds, whereas risks were lower for aquatic plants (12.9%) and fish (6.5%). Mixture toxicity threshold exceedances indicate adverse ecological effects to occur at half of sites (50%). This study illustrates the presence of pesticide mixtures and highlights ecological risks for aquatic organisms in surface waters of protected areas in Germany.

**Supplementary Information:**

The online version contains supplementary material available at 10.1007/s00128-023-03830-5.

## Introduction

With more than 50% of the total land area, agriculture is the biggest land use type in Germany (Federal Agency of Nature Conservation 2014). Here, pesticides are a widespread tool for plant protection, and are often considered essential to enhance yield quality and quantity (Sharma et al. 2019). Pesticides are applied to agricultural fields and can enter nearby waterbodies via, e.g. drift, runoff, or drainage, where they can potentially cause adverse effects on aquatic organisms (Stehle and Schulz 2015a; Knauer 2016). Runoff, e.g. during heavy rainfall events, can produce particularly high, but often transient contamination events (Liess et al. 2021) which require targeted (“event”) sampling to be captured (Stehle et al. 2013).

Due to the potential risks posed to ecosystems, the European Union regulates their use by Regulation (EC) No 1107/2009 (European Union 2009), requiring a registration of pesticides. As part of this registration process, Regulatory Threshold Levels (RTLs; for details see Stehle and Schulz 2015a) are established for different organism groups (e.g. aquatic invertebrates, fish, and plants) and are derived from standardized ecotoxicity test data, which are subsequently applied with so-called uncertainty factors. These RTLs should not be exceeded to ensure that no adverse effects occur in the environment. However, frequent exceedances of RTL have been reported for both agriculturally dominated surface waters (Wolfram et al. 2018; Stehle and Schulz 2015a; Liess et al. 2021) and surface waters in general (Wolfram et al. 2021; Malaj et al. 2014; Stehle et al. 2019). A recent study by Wolfram et al. (2023) demonstrated that surface waters in protected nature conservation areas in Germany are also exposed frequently to pesticide mixtures that might pose considerable risks to aquatic organisms. However, this issue has also been observed in other countries, for instance in surface waters (Ferrario et al. 2017; Bradley et al. 2021), sediment (Barakat et al. 2013), and in biota (Smalling et al. 2013; Gerber et al. 2016).

Protected areas are intended for long-term preservation of nature (e.g. biodiversity) and its resources (e.g. drinking water), hence represent critical areas for safeguarding biodiversity and its value to humankind (European Environment Agency 2020a). Thus, investigating the issue of pesticide surface water exposure and risks for aquatic biodiversity resource provision in protection areas appears an important requirement.

The aim of this study was to analyze and characterize the occurrence of pesticides in a drinking water protection area and a river catchment originating from the biosphere reserve Palatinate Forest. Potential ecotoxicological risks of the detected pesticide mixtures towards aquatic invertebrates, fish, and plants were assessed.

## Methods and Materials

### Monitoring Sites and Sampling

Surface water samples in two regions in southwestern Germany were taken during the main growing season (May 2022 to August 2022). A total of seven stream sites were sampled in the drinking water protection area Hausen near Freiburg harboring a mix of agricultural uses (see Fig. S 1), which provides drinking water for approximately 155,000 people (Kaier 2013), and four sites in the catchment of the river Queich near Landau, originating in the biosphere reserve Palatinate Forest, and flowing through viticulture, and further downstream, vegetable growing areas (see Fig. S 2).The seven surface water monitoring stations in the area of Hausen (FR1–FR7) were established within the drinking water protection area covering all important tributaries of its wells, and its sub-catchments. The four monitoring stations near Landau (LD1–LD4) were established along the river Queich in the biosphere reserve, upstream of any wastewater treatment plant inlet and agricultural areas (LD1), downstream in highly intensified viticulture (LD2) and, further downstream, in an area of fruit and vegetable farming (LD3–LD4). Site LD2 was situated in a tributary of the Queich, shortly before its entry into the main channel. The site was chosen to analyze if contaminants are transported into small tributaries, e.g. via subsurface flows, drainage or atmospheric deposition.

In total, 47 event-driven surface water samples were collected throughout all sites to capture peak pesticide concentrations resulting primarily from run-off events. An event was defined as heavy rainfall (> 10 mm) within < 4 h. For rainfall and temperature data and sampling dates, see Fig. S 3. Two different sampling methods were applied at each monitoring station, event sampling and grab sampling. Event samples were collected with brown glass vessels (540 mL or 1 L) fixed at up to three different heights above the water line (levels; A–C, for details see Fig. S 4), filling automatically with a rising water level following heavy rainfall (Schulz 2001). Approximately 6–18 h following a rainfall event, 15 mL were transferred from the filled glass vessels into 20 mL borosilicate vials (ROTILABO^®^, Carl Roth^®^). The used glass vessels were replaced by clean glass vessels after each event. Grab samples (15 mL) were taken from the center of the stream, 5–10 cm below the water surface, with the vial’s opening facing downstream. All grab samples were taken at the same day during which event samples were collected. Due to short-term fluctuation peaks after heavy rainfall events, higher concentrations are expected in the event samples compared to grab samples. All samples were immediately cooled and transported back to the laboratory, where they were frozen at -18 °C for further analysis. Duplicates were taken of all event and grab samples. In the area of Hausen, four events (E1–E4) were sampled and in the Queich catchment three events were sampled (E1–E3, see Fig. S 3).

### Chemical Analysis

Water samples were analyzed for 89 pesticides which reflect the current-use pesticides that are commonly applied in agricultural crops throughout southwestern Germany (see Table S 1) by high-performance liquid chromatography coupled to triple quadrupole mass spectrometry using a direct injection method (Roodt et al. 2023). Briefly, water samples were centrifuged (16,000 rpm, 10 min) before 350 µL was transferred to an amber-glass vial. The samples were then diluted with 150 µL of methanol containing a mixture of three deuterated internal standards (pirimicarb-D6, thiacloprid-D4 and thiamethoxam-D3) and 0.3% formic acid. Analytical limits of quantification are reported in Table S 1. Quality of results was assessed by monitoring the concentrations of three deuterated internal standards, which were added to each sample at a concentration of 1.0 ng/mL. Internal standard recoveries between 70 and 120% were considered acceptable. In addition, two laboratory blank samples were prepared alongside the field samples and revealed no detectable compounds.

### Ecotoxicological Effect Thresholds

Effect thresholds, represented by tier-1 RTL (see Stehle and Schulz 2015b), were derived for the three species groups aquatic invertebrates, fish, and aquatic plants. RTLs denote thresholds above which adverse effects are expected to occur in aquatic ecosystems (Stehle et al. 2013; Wolfram et al. 2021). They are based on standardized acute ecotoxicity data (e.g. 96 h-EC_50_* Daphnia magna*) for each organism group that is subsequently divided by uncertainty factors (100 for fish, invertebrates, 10 for algae) yielding thresholds at which adverse ecological effects are known to occur (Schäfer et al. 2012; Beketov et al. 2013). RTLs were taken from Wolfram et al. (2023) or, in case none was available for a specific combination of substance and species group, were calculated from ecotoxicity endpoints (e.g. EC_50_) from the Pesticide Properties DataBase (PPDB; Lewis et al. 2016) and divided by uncertainty factors according to Stehle and Schulz (2015a). Effect data from the PPDB were only used if assigned with high quality indicators (A4–A5), signifying their use in EU regulatory risk assessments. Of 89 pesticides analyzed, 76, 75, and 81 could be assigned with RTL for aquatic invertebrates, fish, and aquatic plants, respectively (see Table S 2). Consequently, 83.7%, 81.7% and 89.1% of detections (n = 1,962) could be ecotoxicologically assessed by comparing the measured environmental concentration with a tier-1 RTL, yielding measured environmental concentration to threshold ratios (M/R). Environmental risks of co-occurring pesticides in samples were also assessed assuming concentration addition (Backhaus et al. 2004), yielding sum(M/R), and were based on the highest risk observed in either event or grab samples (see Wolfram et al. 2023).

### Data Analysis

We analyzed the number of quantified pesticides per sample, the sum concentration per sample, the combined risk per sample sum(M/R) and the relative risk contribution of individual pesticides to the sum(M/R). Land use (European Environment Agency 2020b) and crop distribution data (Blickensdörfer et al. 2021) were used to characterize upstream catchments and a corridor of 300 m along the upstream flowline (Bunzel et al. 2014; see Table S 3, Table S 4). Similarity of pesticide detections between sampling sites and events (presence vs. absence) were expressed via Jaccard distances (Hancock 2014). All calculations and figures were prepared using R (R base: Ver. 4.0.3, 64-bit, Windows 10; R Core Team 2019) and QGIS (Ver. 3.26.1 Buenos Aires, 64-bit, Windows 10; QGIS.org 2021).

## Results and Discussion

During the first run-off event (E1) in the area of Hausen, up to a maximum of 43 substances, comprised of 25 fungicides, eleven herbicides, and seven insecticides, were detected simultaneously in one individual sample (FR7; see Fig. S 5). FR7 is located at the ditch Burggraben, a small catchment with predominantly maize, grains, and vegetables grown in its near surrounding (see Table S 3). In Landau, a maximum of 38 substances (20 fungicides, twelve herbicides, and six insecticides) were detected simultaneously during E1 at LD4, which captures both intensive upstream viticulture and surrounding intensive fruits and vegetable growing areas (see Fig. S 5, Table S 3). On average, 32 (n = 21) and 21 (n = 10) pesticides were detected in the area of Hausen and the Queich catchment during all events, respectively.

**Fig. 1:**
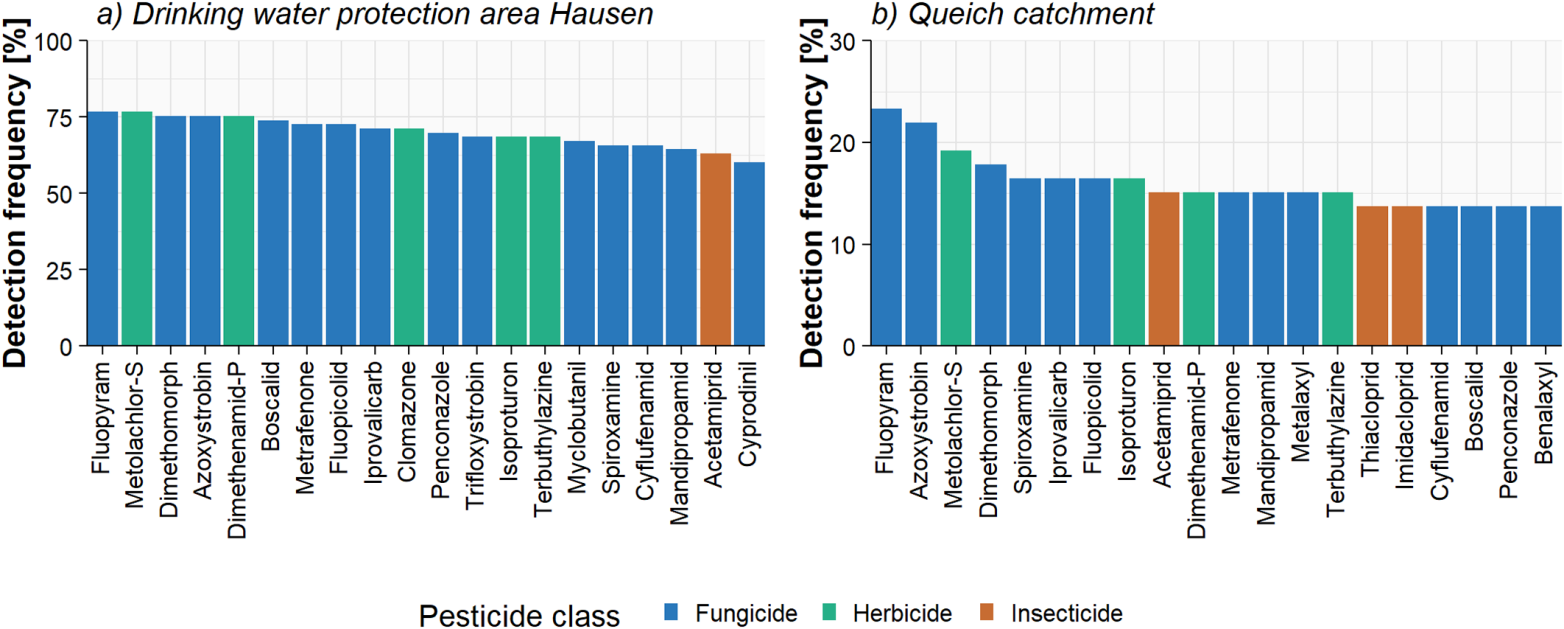
Detection frequencies of all samples for the 20 most frequently detected substances for the drinking water protection area Hausen (**a**) and the Queich catchment (**b**) that were above their limit of detection at least once

In total, 28 substances were detected with a detection frequency > 50% in the area of Hausen and the Queich catchment (mean detection frequency = 10.1%), with the detection frequencies being significantly higher for Hausen (mean detection frequency = 35.5%, *p*-value < 0.001). The fungicide fluopyram was found with the highest detection frequencies of 76.7% and 23.3% in the area of Hausen and the Queich catchment, respectively (Fig. 1). Mainly fungicides and herbicides were among the most frequently detected pesticides, with the neonicotinoid insecticide acetamiprid being the only insecticide detected within the 20 most frequently detected substances in the area of Hausen. In addition to acetamiprid, two other insecticides were found within the 20 most frequently detected substances in the Queich catchment. Insecticides generally occurred less frequently in line with their distinct application patterns and short environmental half live times, contrasting the pre-emptive and repeated applications of most herbicides and fungicides (Bundesamt für Verbraucherschutz und Lebensmittelsicherheit 2019). Acetamiprid is still approved in the EU whereas thiacloprid is no longer approved since January 2020 (European Commission 2020). Thiacloprid was nonetheless detected at all monitoring sites, reaching a maximum concentration of 31.6 ng/L. Illegal applications appear to be an unlikely explanation in light of its occurrence at multiple sites, the frequent presence thus rather indicates remobilization in nearby fields, questioning the assumed fast degradation times (1–2 weeks) in soils (Lewis et al. 2016).

Event samples were overall better in capturing pesticide mixtures and peak concentrations compared to grab samples (Fig. S 5, Fig. S 6). At LD1, during E1, a sum concentration of 118.1 ng/L comprising ten fungicides, one insecticide, and seven herbicides was observed (Fig. S 6), although the site is situated within the biosphere reserve and upstream of any wastewater treatment plant inlet or agricultural areas (minor cultivation of fruits, see Table S 3). Previous investigations (*pers. comm.* S. Stehle) have also occasionally detected multiple pesticides in other surface waters within the biosphere reserve despite lacking any direct pesticide sources. Medium-range atmospheric transport and residential pesticide use may be responsible for this observation, which has already been observed as being relevant entry pathways in other remote or protected surface waters globally (Ackerman et al. 2008; Smalling et al. 2013; Daly et al. 2007; Hageman et al. 2006; Kaiser 2011). Furthermore, prolonged droughts and severe weather events (e.g. heavy rainfall) could lead to dry-deposited pesticides being transported (Messing et al. 2013) and washed-off in remote areas, resulting in mixtures of contaminants. Yet, subsequent events E2 and E3 only showed two and three pesticides at very low sum concentrations of 2.2 and 3.8 ng/L at LD1, respectively. Future work should investigate potential entry pathways, e.g. medium-range atmospheric transport, which could expose protected and assumed-to-be pristine environments to pesticide mixtures, as evidenced in an evaluation of a large dataset for natural conservation areas in Saxony, Germany (Wolfram et al. 2023). LD2 is a secondary branch of the river Queich with minimal flow similar to a wetland area. During E1 and E3, no contaminants were detected at LD2, whereas during E2, a total number of 25 substances with a sum concentration of 281 ng/L was found (Fig. S 5, Fig. S 6). One assumption is that contaminants from E1 did not yet reach LD2 due to its very low flow speed, i.e. only being detected during E2 which was three days after E1. The similarity between substances detected at LD2 (E2) and the other sites in the Queich catchment at E1 (see Fig. S 7) adds some support to this assumption. The sampling site FR1 was largely dried out, thus, no regular sampling was possible. However, > 30 substances were found with a sum concentration of 0.9 µg/L after E3. For the Queich catchment, the highest sum concentration of 1.6 µg/L was observed at LD4 during E1 (Figure S6). Overall, higher concentrations were observed in the area of Hausen. The highest sum concentration of 6.3 µg/L was observed at FR7 for E2 (Fig. S 6).

Pesticide mixtures among sites were generally similar in terms of substances found, as detailed by their Jaccard distance (see Fig. S 7, Fig. S 8). In the area of Hausen, pesticide mixtures were overall similar between all sites (mean = 63.2%), whereas in the Queich catchment, the similarity was substantially lower (mean = 35.7%). As such, the varying land-use and land-cover along the larger Queich catchment resulted in clear differences in the observed pesticide mixtures, highlighting presence of varying pollution sources along this course (Fig. S 7). In contrast, the similarity of detected compounds between all sites in the area of Hausen indicates a more uniform distribution of contaminants within the drinking water protection area, despite the differences in crop composition, suggesting a diffuse entry of pesticides throughout the whole area (Fig. S 8). To limit the transport of contaminants into surface waters would require a comprehensive approach, e.g. strict protection of riparian zones to curb non-point source pollution or prohibiting pesticide use within these tributaries of the wells. The drinking water protection area of Hausen may be indicative for current land-use challenges in the federal state of Baden-Wurttemberg, where approximately 32% of all agricultural areas are within drinking water protection areas, whereas in other federal states, e.g. Bavaria and Lower Saxony, these shares are substantially lower (3.8% and 7.6%; *pers. comm.* L. Eichler).

### Environmental Risks

**Fig. 2:**
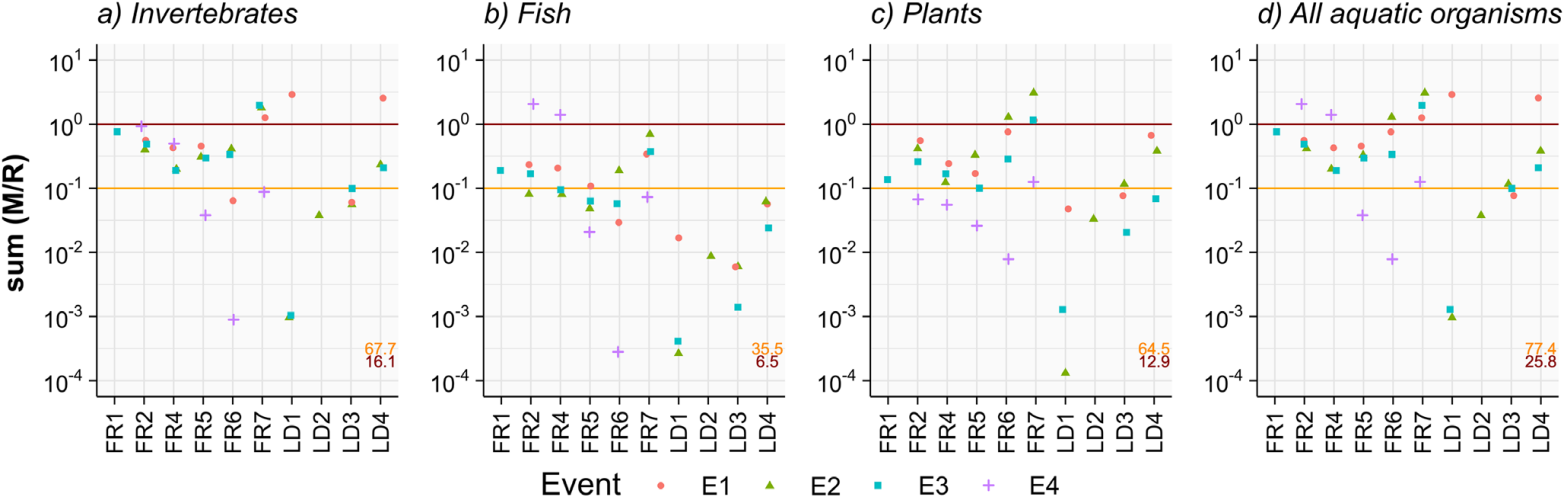
sum(M/R) for fish (**a**), aquatic invertebrates (**b**), aquatic plants (**c**) and combined for all three species groups (**d**) per monitoring site and for all three (Queich catchment; LD) or four (area of Hausen; FR) events. Percentages of sum(M/R) > 1 and > 0.1 are annotated in red and orange, respectively

Risks were highest for aquatic invertebrates (Fig. 2) with 16.1% of sum(M/R) > 1, indicating acute risks, and 67.7% of sum(M/R) > 0.1, a threshold at which reduction of family richness can occur already for single substances (see Stehle and Schulz 2015a). Risks were lower for fish and aquatic plants with 6.5% and 12.9% of sum(M/R) > 1, respectively, and 35.5% and 64.5% of sum(M/R) > 0.1, respectively (Fig. 2). Similar patterns in benchmark exceedances were observed by Nowell et al. (2021), Wolfram et al. (2021), and Malaj et al. (2014) indicating highest risks for invertebrates in U.S. and European surface waters, respectively. Overall, in 25.8% of all events (n = 73), the sum(M/R) exceeded 1 for at least one species group, potentially impairing their associated ecological functions. Event samples were overall better at capturing transient peaks, and thus, describing the acute ecological risks more accurately: on average, event sample sum(M/R) were higher by a factor of 4.2, 2.8, and 3.1 for aquatic invertebrates, fish, and plants, respectively (Fig. S 9).

The first runoff event within the biosphere reserve (location LD1) caused significant risk for aquatic invertebrates with a sum(M/R) of 2.9, despite its predominantly natural land-use, i.e. grassland (see Table S 3). In the area of Hausen, three out of the six monitoring stations showed at least one sum(M/R) > 1, and all stations showed sum(M/R) > 0.1. Hence, risk profiles were overall similar between the sites in the area of Hausen, in line with the high similarity of observed mixtures, which likely results from the comparable agricultural intensity along all monitored streams (see Table S 3, Table S 4).

Environmental risks for each species group were driven by distinct contaminants (see Fig. S 10), with fungicides driving risks for fish, and insecticides and herbicides for aquatic invertebrates and plants. For fish, the fungicides trifloxystrobin and pyraclostrobin had median contributions to the sum(M/R) of 37% and 25.5% in 55 and 8 samples, respectively, underscoring their regular presence at ecologically relevant concentrations in the observed mixtures, and their important contribution to risks for fish (Fig. S 10). For aquatic invertebrates, the insecticides chlorantraniliprole (n = 24, not belonging to the top-20 group with regard to detection frequency) and imidacloprid (n = 16), and the fungicide cyprodinil (n = 44), regularly drove the observed risks in 53.8%, 18.6%, and 20.6%, respectively. Imidacloprid recently lost its approval in December 2020, although its grace period was extended until June 2022 (European Commission 2021), which allowed farmers to use any remaining imidacloprid products. Similarly, Brühl et al. (2021) concluded that the high incidence of thiacloprid in insects in nature conservation areas was due to its ending grace period which gave the last opportunity for farmers to use their remaining products. For aquatic plants, two herbicides (dimethenamid-P, metolachlor-S) and one fungicide (spiroxamine) primarily drove the risk with a median contribution to the sum(M/R) > 12%, respectively. While these substances contributed most to sum(M/R) for respective species groups, it was the mixtures that overall resulted in the pronounced risks, i.e. requiring on average 5 and 7 substances to explain 90% and 95% of sum(M/R), respectively. Previous work highlighted how environmental risks of pesticide mixtures can be often defined by single contaminants, particularly in the case of insecticides affecting aquatic invertebrates (Wolfram et al. 2019) and herbicides affecting primary producers (Wolfram et al. 2023). In this study, however, the combination of low analytical limits, event-driven samples, and the diverse agricultural landscape revealed mixtures with multiple relevant toxicants acting jointly on these aquatic ecosystems.

Safeguarding surface waters that feed groundwater aquifers from chemical pollution will become even more critical in the future, especially in response to current climatic changes, which are expected to affect seasonal water availability particularly during spring and summer (Hajek and Knapp 2022). Lateral transport of mobile pesticides can result in direct contamination of groundwater aquifers (Muneer Ahmad Malla et al. 2021), contaminating drinking water supplies for human consumption and adversely affecting susceptible microfauna therein (Bexfield et al. 2021). In this context, of the 20 most commonly detected pesticides, three are classified with a high GUS-leachability potential (> 2.8), ten with transition states (1.8–2.8), and seven having low leachability (< 1.8; Lewis et al. 2016), describing their potential to move into the groundwater. The hyporheic zone also provides vital ecological functions, e.g. nutrient and detritus turnover, filtration, degradation of contaminants, which positively affect both surface- and groundwater quality (Griebler and Avramov 2015). Therefore, the environmental risks detailed here are concerning, given the importance of drinking water protection areas, which is also recognized by the Sustainable Use Directive currently discussed in the EU (Food Safety 2023). Substantial mitigation strategies will be necessary in the future to reach these ambitious, yet necessary targets.

For the Landau sampling sites, environmental risks generally increased along the course of the river for all species groups (Fig. 2), with the exception of E1 at LD1 (see above). Lotic systems are known integrators of contaminants due to their flow direction throughout the landscape, leading to higher exposure to pesticide inputs (Wolfram et al. 2019; Stehle et al. 2019; Liess et al. 2021). The Queich catchment has mostly natural upstream reaches located in the Palatinate Forest with nearly no direct pesticide sources. However, it remains uncertain how effectively initiatives like the Sustainable Use Directive for pesticides (Food Safety 2023) will protect such sensitive areas. Firstly, pronounced ecological risks were found at LD1 where agricultural and anthropogenic influence is very low, indicating that pesticides may have been transported atmospherically from westward agricultural areas. Secondly, recent analyses showed how pesticides are transported into surface waters of nature conservation areas via fluvial inflows (Wolfram et al. 2023). Thus, other sensitive areas further downstream the river Queich, e.g. Natura 2000 areas, are likely exposed to mixtures of contaminants simply due to their hydrological connection to unprotected areas (Wolfram et al. 2023).

The present study reveals the presence of pesticide mixtures in surface waters of protected areas in Germany at concentrations of environmental concern, particularly for aquatic invertebrates, which other studies have also observed in remote areas in both Europe and United States (Wolfram et al. 2021; Nowell et al. 2018). In both sampling regions, event samples performed better at capturing pesticide mixtures after heavy rainfall events compared to grab samples. However, it is currently unclear, whether potential chronic background concentrations of pesticides may have affected the pesticide mixtures and their sum concentrations in this study. Thus, subsequent investigations will focus on determining the presence and complexity of pesticide mixtures to characterize potential long-term impairments of these vital aquatic ecosystems.

### Electronic supplementary material

Below is the link to the electronic supplementary material.

Supplementary Material 1
