# Supplementary material for: Pesticide Mixtures in Surface Waters of Two Protected Areas in Southwestern Germany
Source: Bull Environ Contam Toxicol. 2023 Dec 12;112(1):10. doi: 10.1007/s00128-023-03830-5 (PMC10716062; doi:10.1007/s00128-023-03830-5)
Supplement: Supplementary file 1 — Supplementary Material 1 [file 128_2023_3830_MOESM1_ESM.docx]

**Supplementary Material**

**SI Figures**


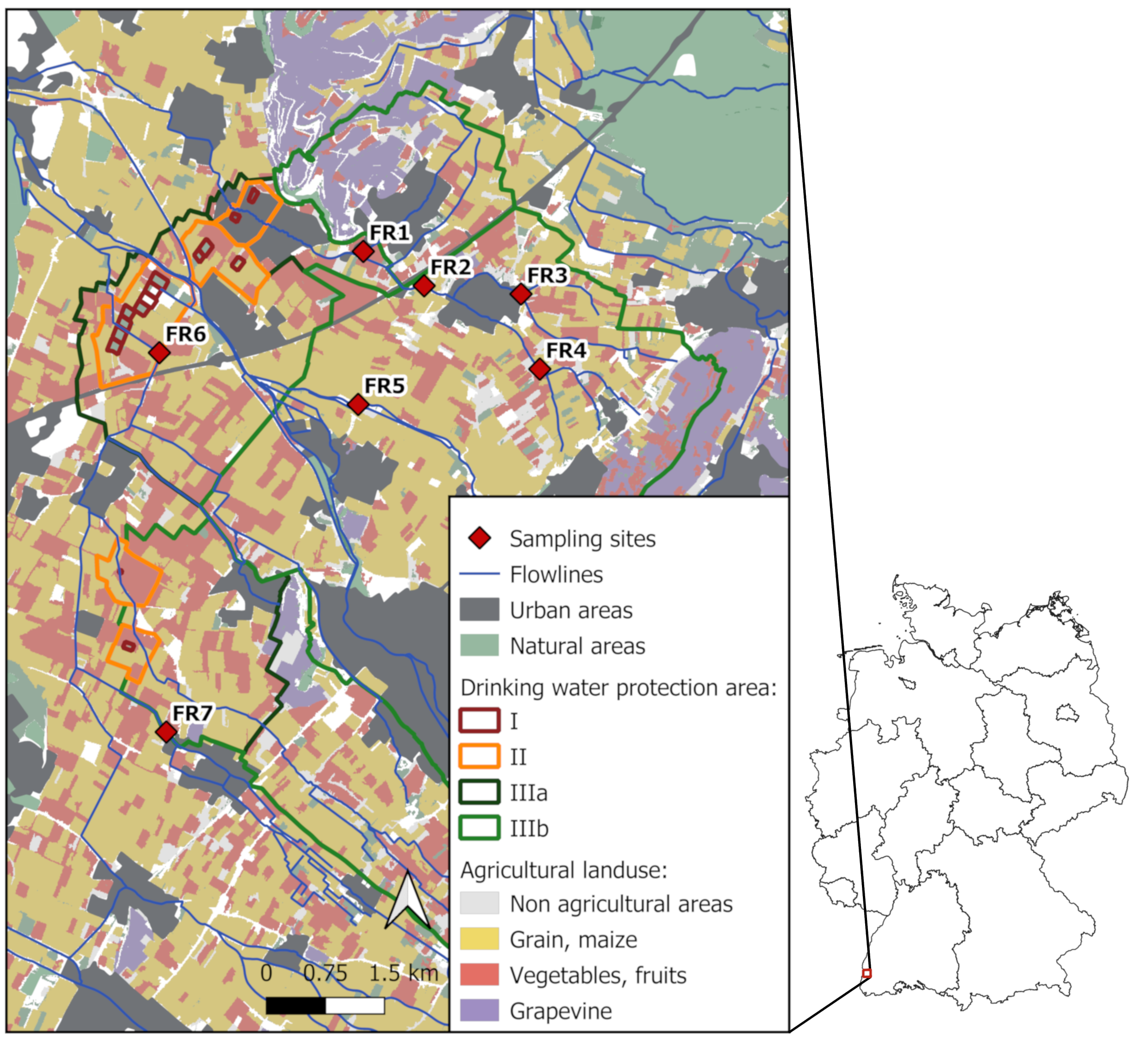


**Fig. S 1** Sampling sites (FR1–FR7) within the drinking water protection area near Hausen (Freiburg) and its sub-catchments in southwestern Germany, with respective land use.


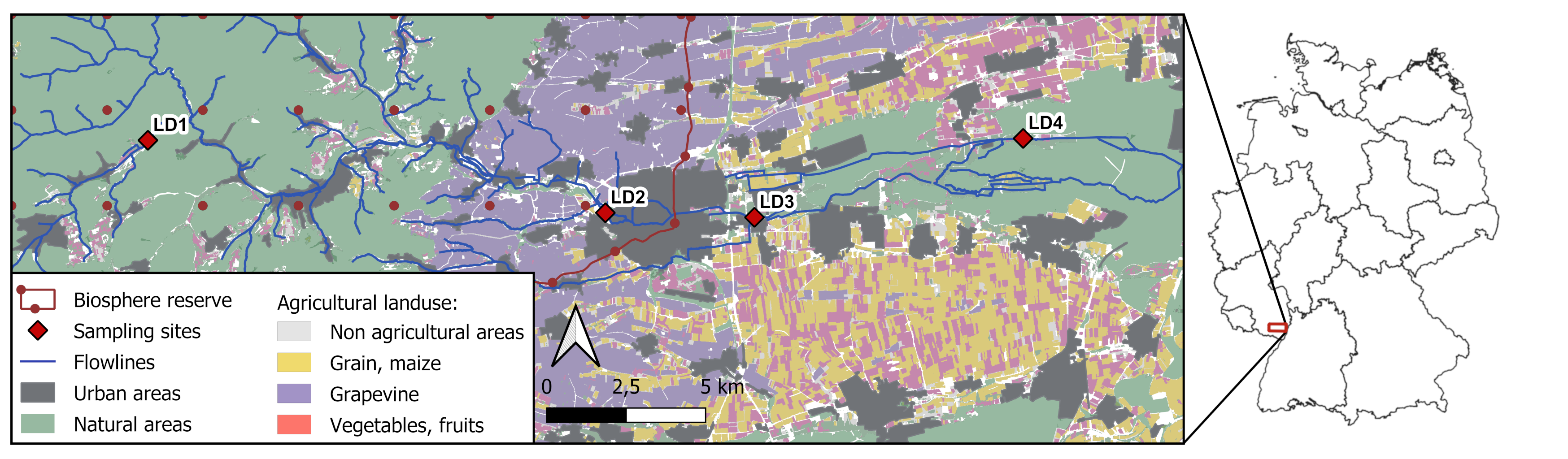


**Fig. S 2** Sampling sites (LD1–LD4) within the Queich catchment (Landau) in southwestern Germany, with respective land use and the biosphere reserve Palatinate Forest.


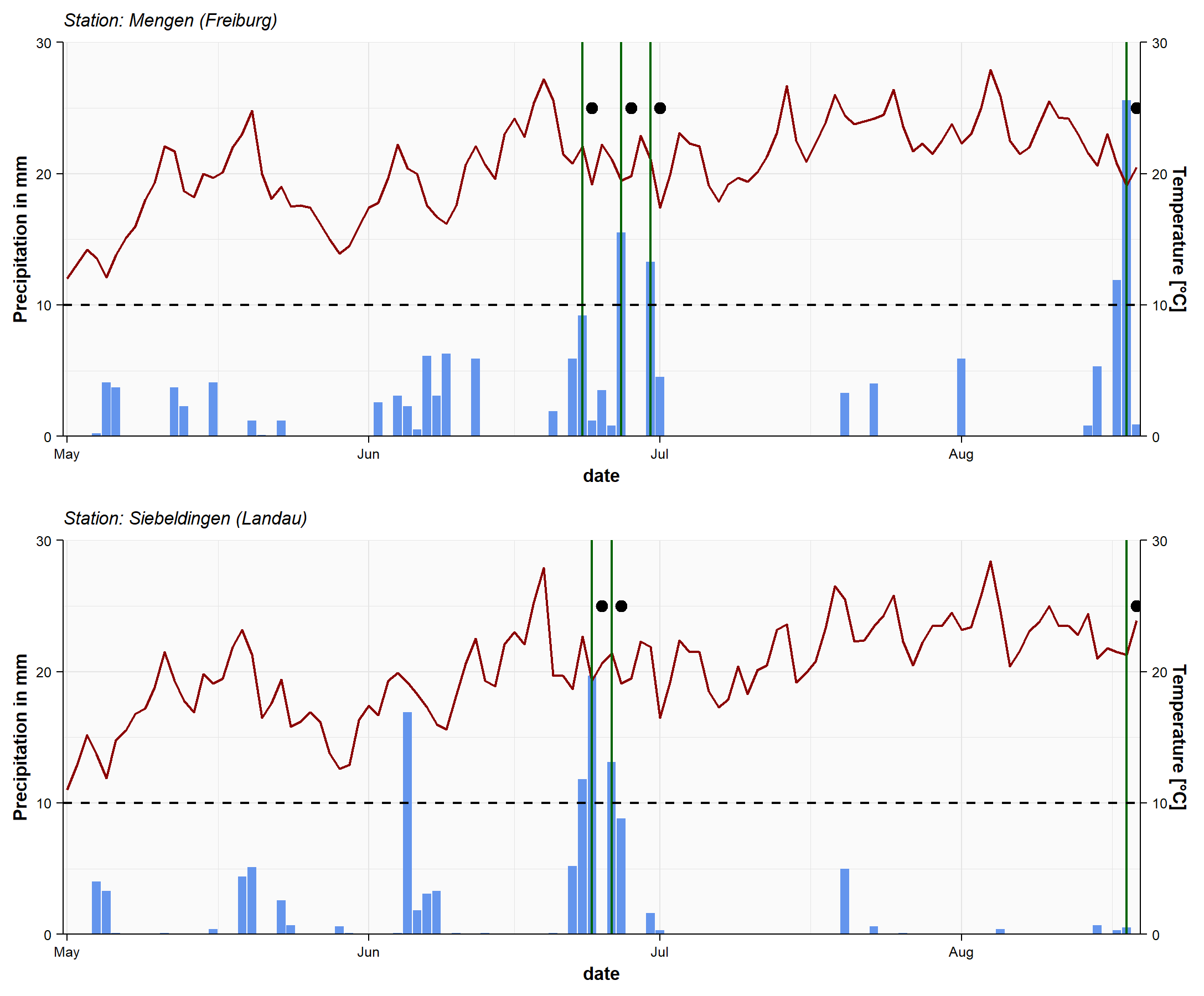


**Fig. S 3** Daily precipitation (mm) and average daily temperature (°C) for (a) the weather station Mengen (German Weather Service) within the drinking water protection area near Hausen (Freiburg) and (b) the weather station Siebeldingen (German Weather Service) within the Queich catchment (Landau). The dashed line indicates the threshold for a heavy rainfall event. Note that in early June, the rainfall in Siebeldingen was highly localized with no rainfall occurring in Landau, thus no samples were collected. Event samples are represented by a black dot.


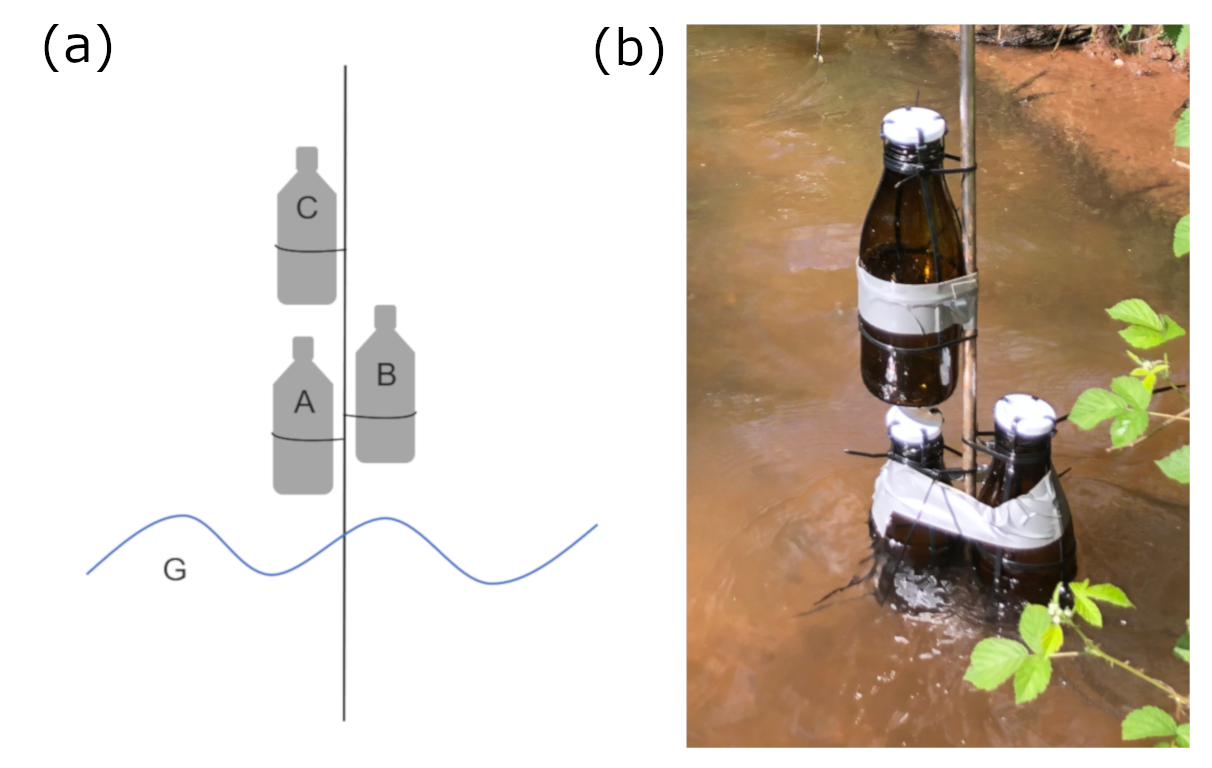


**Fig. S 4** (a) Schematic depiction of an event sampler displayed with three inert brown glass vessels (540 mL or 1 L) assigned to three different levels above the waterline (A–C) and a grab sample (G). (b) Photographic image of an event sampler installed in the drinking water protection area near Hausen, Freiburg.


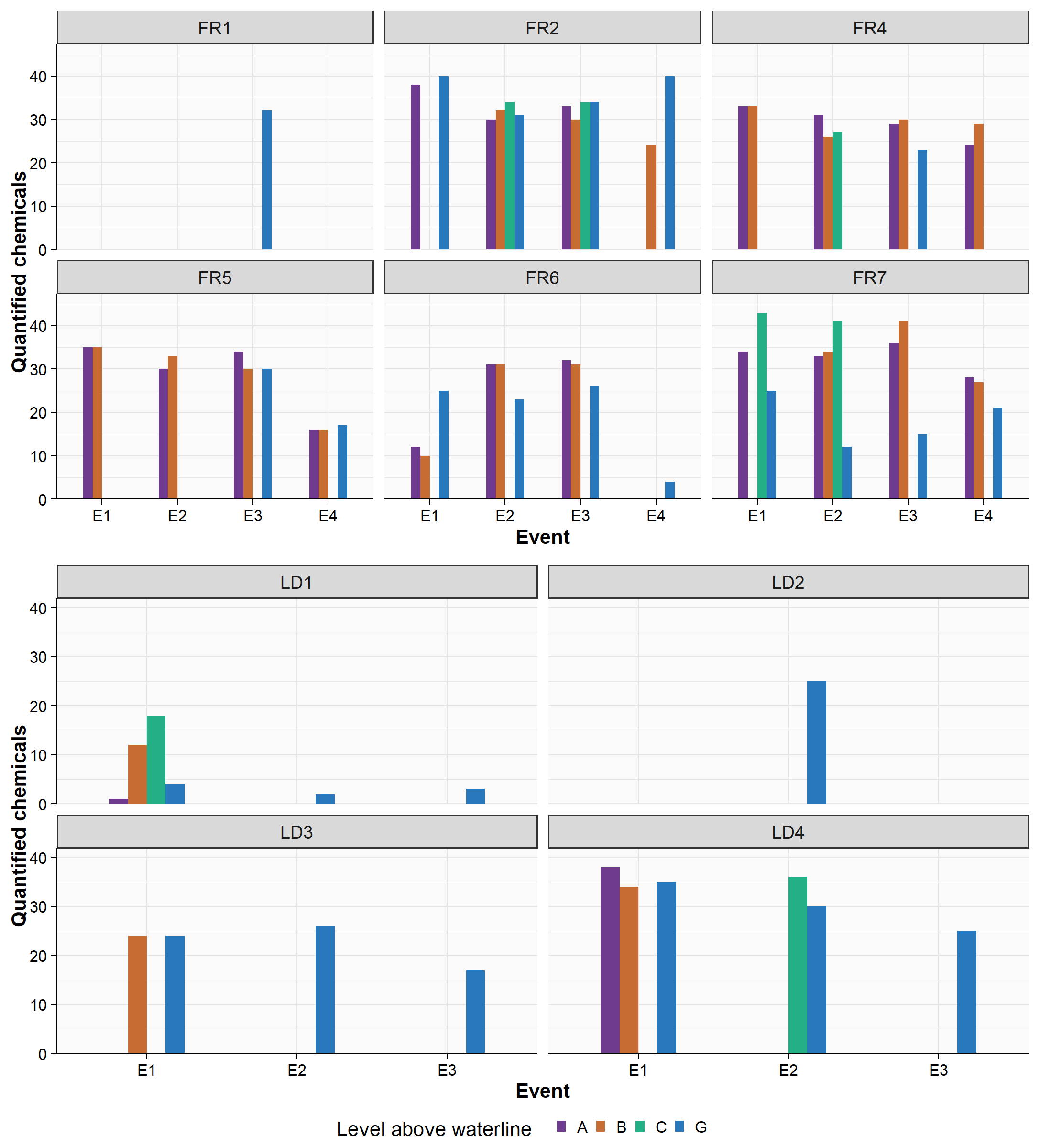


**Fig. S 5** Number of simultaneously quantified substances per sample found in the three different levels above the waterline (A–C) and in the grab sample (G) for each event (E1–4) and the different monitoring sites for the area of Hausen (FR1–FR7) and the Queich catchment (LD1–LD4).


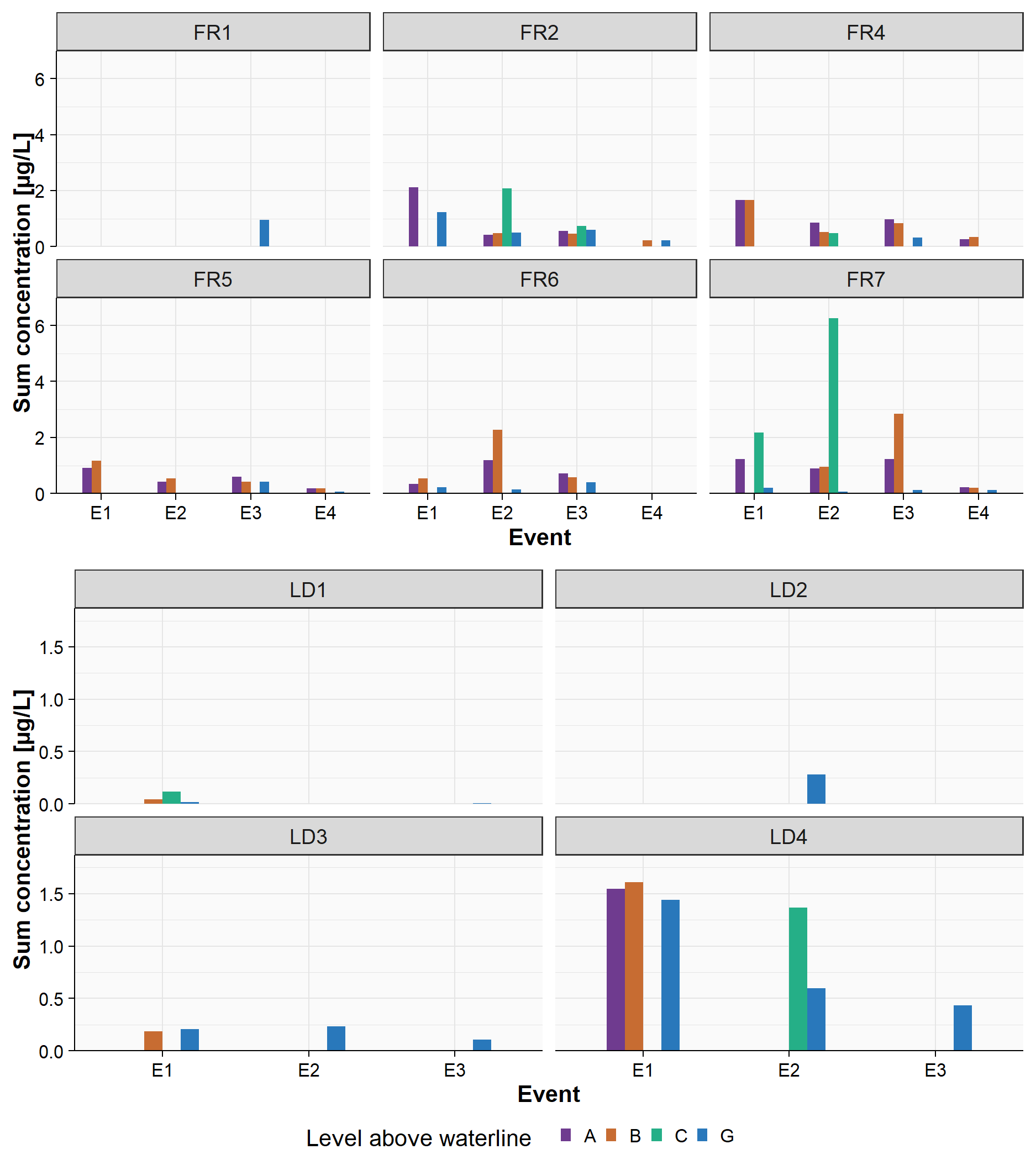


**Fig. S 6** Sum concentrations per sample of the three different levels above the waterline (A–C) and grab sample (G) for each event (E1–4) for the different monitoring sites for the area of Hausen (FR1–FR7) and the Queich catchment (LD1–LD4).


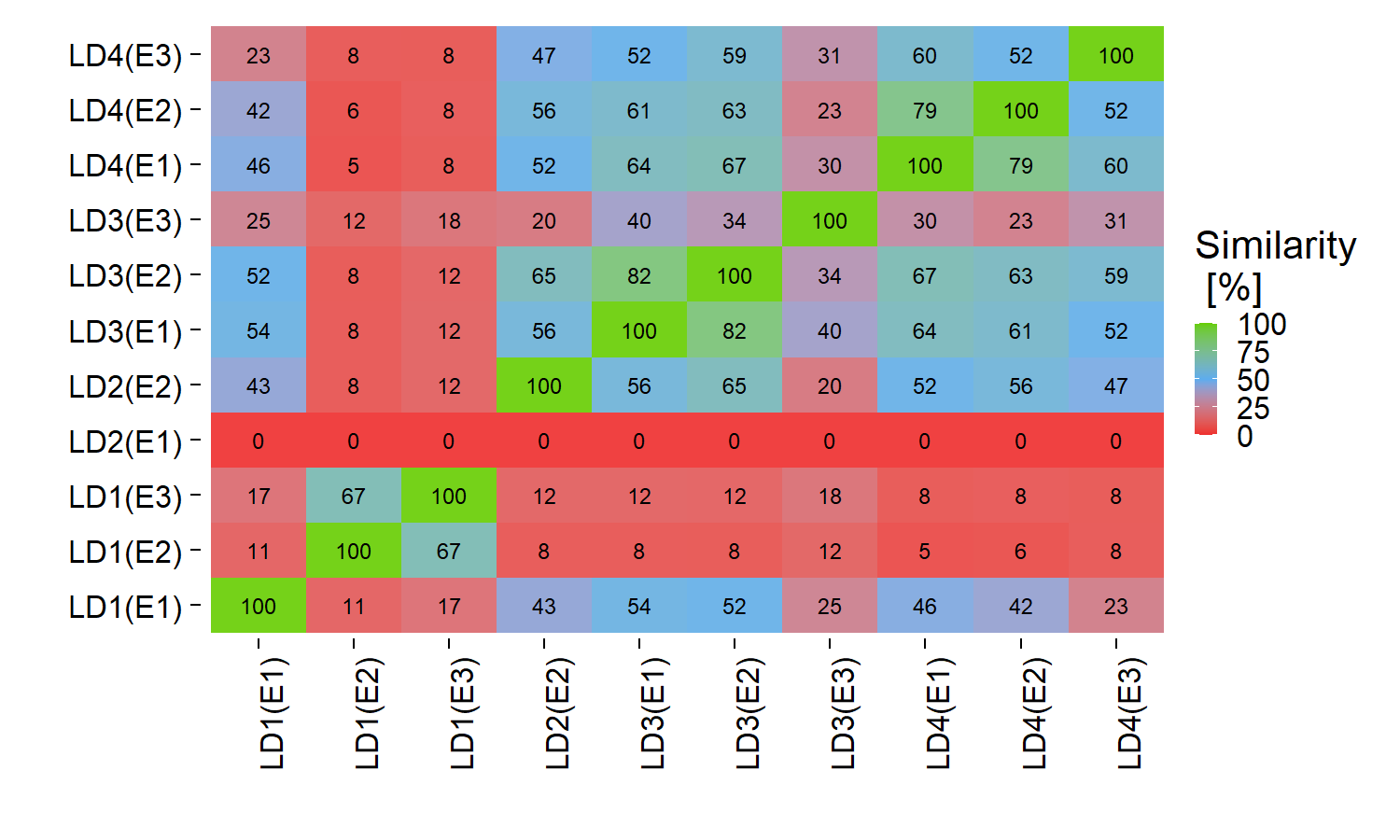


**Fig. S 7** Heat map showing similarities substance detections at each monitoring site and event (E1–E3) of the Queich catchment (LD1–LD4). Similarities between samples are expressed via Jaccard similarities in percent (1 - Jaccard distance).


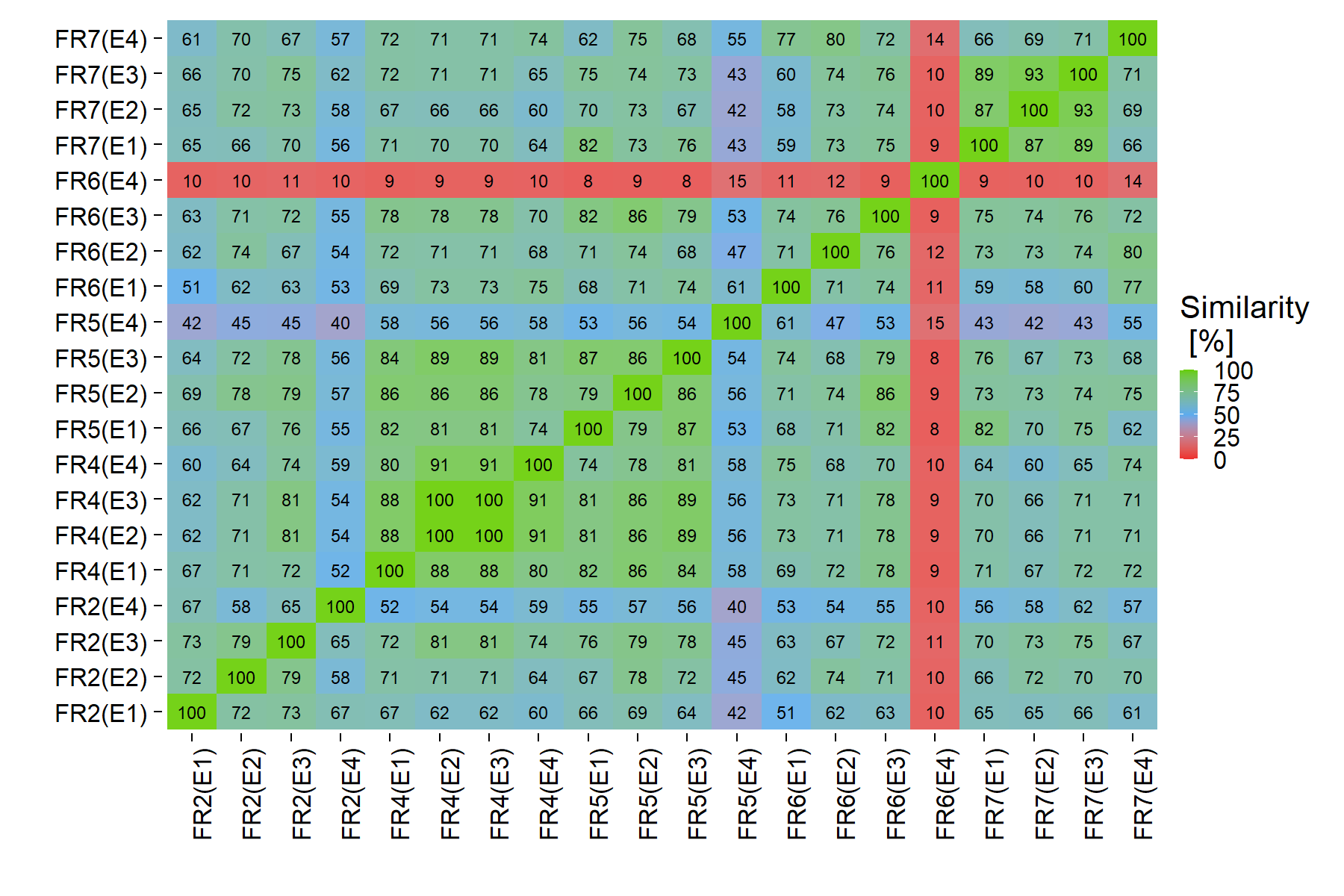


**Fig. S 8** Heat map showing similarities of substance detections at each monitoring site and event (E1–E4) for the area of Hausen (FR1–FR7). Similarities between samples are expressed via Jaccard similarities in percent (1 - Jaccard distance).


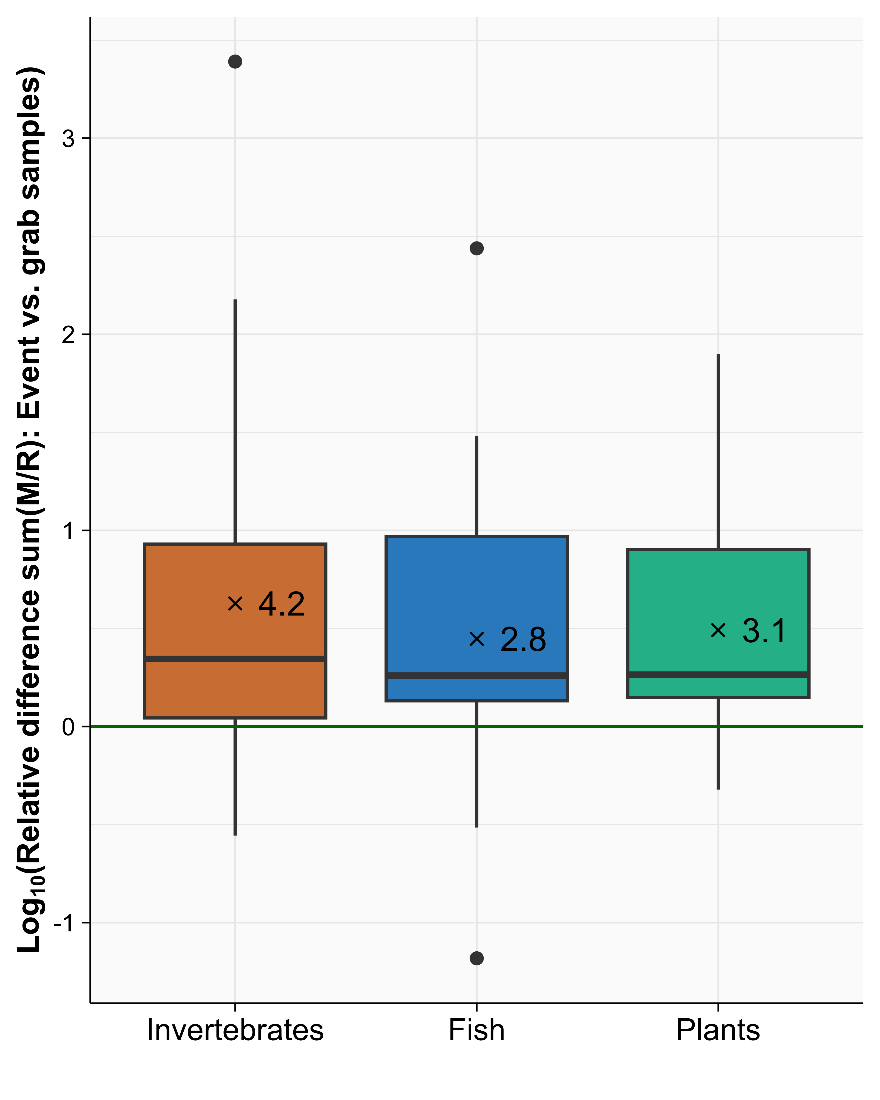


**Fig. S 9** Relative difference in sum(M/R) between event and grab samples for the three aquatic groups invertebrates, fish, and plants on a log_10_-scale. Higher values indicate that event samples described higher environmental risks compared to grab samples. The average relative difference is annotated in black and symbolized by a cross.


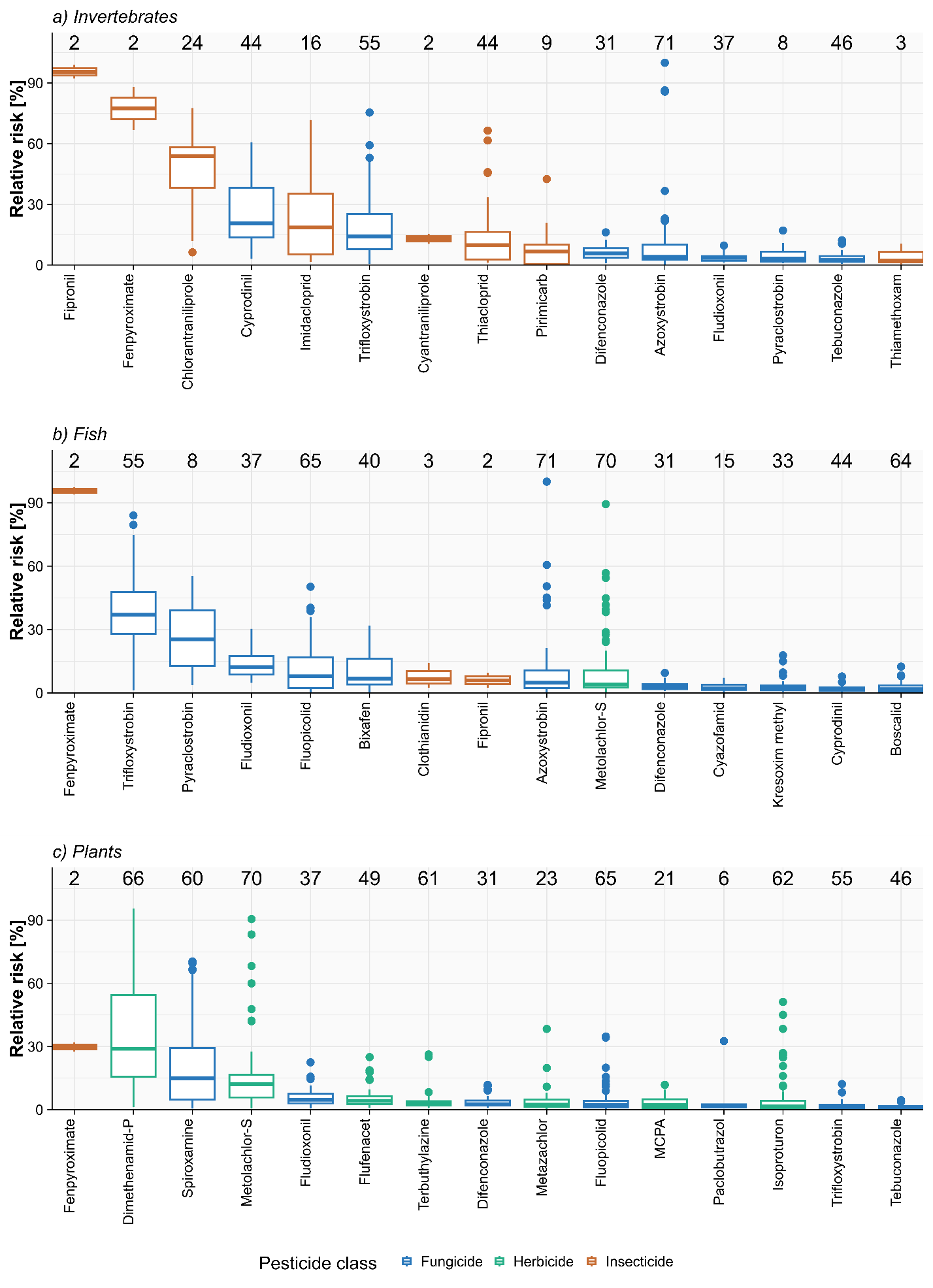


**Fig. S 10** Substances` relative sum(M/R) contribution per sample (%) for (a) aquatic invertebrates, (b) fish, and (c) aquatic plants. The number of samples to which each substance contributed is annotated at the top. A maximum of 73 samples (site × event) was possible.

**SI Tables**

**Table S 1** Analytical limits of quantification (LOQ) for the compounds monitored in the water samples in the area of Hausen and the Queich.

| **Compound** | **LOQ [ng/L]** |
| --- | --- |
| 2,4-D | 100 |
| Acetamiprid | 0.3 |
| Aminopyralid | 10 |
| Azoxystrobin | 0.3 |
| Benalaxyl | 1 |
| Bentazone | 0.6 |
| Bixafen | 1 |
| Boscalid | 2 |
| Bromoxynil | 10 |
| Carfentrazone-ethyl | 10 |
| Chlorantraniliprole | 2 |
| Chloridazon | 0.3 |
| Chlortoluron | 0.3 |
| Clomazone | 0.3 |
| Clothianidin | 0.6 |
| Cyantraniliprole | 1 |
| Cyazofamid | 0.6 |
| Cyflufenamid | 1 |
| Cymoxanil | 2 |
| Cyprodinil | 1 |
| Difenconazole | 10 |
| Diflufenican | 2 |
| Dimethenamid-P | 0.3 |
| Dimethoate | 0.3 |
| Dimethomorph | 1 |
| Dimoxystrobin | 0.3 |
| Epoxiconazole | 2 |
| Ethofumesate | 2 |
| Etofenprox | 50 |
| Fenoxycarb | 1 |
| Fenpropimorph | 1 |
| Fenpyroximate | 5 |
| Fipronil | 1 |
| Flazasulfuron | 0.6 |
| Flonicamid | 5 |
| Florasulam | 2 |
| Fludioxonil | 2 |
| Flufenacet | 0.3 |
| Fluopicolid | 0.6 |
| Fluopyram | 0.3 |
| Flupyradifurone | 1 |
| Fluroxypyr | 50 |
| Flurtamone | 0.3 |
| Foramsulfuron | 2 |
| Hexythiazox | 2 |
| Imidacloprid | 0.6 |
| Iprovalicarb | 0.3 |
| Isoproturon | 0.3 |
| Kresoxim methyl | 0.6 |
| Mandipropamid | 0.6 |
| MCPA | 2 |
| Mecoprop | 50 |
| Metalaxyl | 0.3 |
| Metamitron | 0.6 |
| Metazachlor | 0.3 |
| Metconazole | 5 |
| Methiocarb | 0.6 |
| Metobromuron | 0.3 |
| Metolachlor-S | 0.3 |
| Metrafenone | 2 |
| Metsulfuron-methyl | 1 |
| Myclobutanil | 1 |
| Napropamide | 0.6 |
| Paclobutrazol | 2 |
| Penconazole | 1 |
| Pencycuron | 0.6 |
| Picloram | 10 |
| Picoxystrobin | 0.3 |
| Pirimicarb | 0.3 |
| Prochloraz | 1 |
| Propamocarb | 0.3 |
| Propaquizafop | 5 |
| Propyzamide | 0.3 |
| Prosulfocarb | 0.6 |
| Pymetrozine | 0.6 |
| Pyraclostrobin | 1 |
| Pyrimethanil | 5 |
| Quinmerac | 0.3 |
| Spinosad A | 10 |
| Spinosad D | 50 |
| Spiroxamine | 0.3 |
| Tebuconazole | 10 |
| Tebufenozide | 0.3 |
| Terbuthylazine | 0.6 |
| Thiacloprid | 0.3 |
| Thiamethoxam | 0.3 |
| Tribenuron-methyl | 1 |
| Trifloxystrobin | 0.6 |
| Tritosulfuron | 10 |

**Table S 2** List of detected compounds monitored in the water samples in the area of Hausen and the Queich catchment with their respective CAS, pesticide class, RTL for aquatic invertebrates, fish, aquatic plants, and their corresponding detection frequency.

| **Compound** | **CAS** | **Pesticide class** | **RTL_inv_** | **RLT_fish_** | **RTL_plant_** | **Det. Frequency[%]** |
| --- | --- | --- | --- | --- | --- | --- |
| Acetamiprid | 135410-20-7 | Insecticide | 498 | 1000 | 100 | 78.08 |
| Aminopyralid | 150114-71-9 | Herbicide | 1000 | - | 3000 | 5.48 |
| Azoxystrobin | 131860-33-8 | Fungicide | 1.3 | 4.7 | 9.8 | 97.26 |
| Benalaxyl | 71626-11-4 | Fungicide | 5.9 | 37.5 | 240 | 47.95 |
| Bentazone | 25057-89-0 | Herbicide | 1325 | - | 490 | 1.37 |
| Bixafen | 581809-46-3 | Fungicide | 12 | 0.95 | 9.7 | 54.79 |
| Boscalid | 188425-85-6 | Fungicide | 53.3 | 27 | 375 | 87.67 |
| Chlorantraniliprole | 500008-45-7 | Insecticide | 0.04 | - | - | 32.88 |
| Chloridazon | 1698-60-8 | Herbicide | 1320 | 413 | 60 | 6.85 |
| Chlortoluron | 15545-48-9 | Herbicide | 670 | 77 | 2.4 | 9.59 |
| Clomazone | 81777-89-1 | Herbicide | 5.7 | 155 | 3400 | 83.56 |
| Clothianidin | 210880-92-5 | Insecticide | 0.21472 | 0.29 | 5500 | 4.11 |
| Cyantraniliprole | 736994-63-1 | Insecticide | 0.0947 | 24 | 118 | 2.74 |
| Cyazofamid | 120116-88-3 | Fungicide | 1.9 | 5.6 | 2.7 | 20.55 |
| Cyflufenamid | 180409-60-3 | Fungicide | 10 | 10.4 | - | 79.45 |
| Cyprodinil | 121552-61-2 | Fungicide | 0.33 | 13.5 | 122 | 60.27 |
| Difenconazole | 119446-68-3 | Fungicide | 1.5 | 11 | 3.2 | 42.47 |
| Dimethenamid-P | 163515-14-8 | Herbicide | 27 | 260 | 0.6 | 90.41 |
| Dimethoate | 60-51-5 | Insecticide | 20 | 302 | 9040 | 6.85 |
| Dimethomorph | 110488-70-5 | Fungicide | 120 | 34 | 100 | 93.15 |
| Dimoxystrobin | 149961-52-4 | Fungicide | 0.394 | 0.434 | 1.7 | 2.74 |
| Epoxiconazole | 135319-73-2 | Fungicide | 0.625 | - | 0.78 | 2.74 |
| Ethofumesate | 26225-79-6 | Herbicide | 135.2 | 109.2 | 390 | 4.11 |
| Fenoxycarb | 79127-80-3 | Fungicide | 3.5 | 6.6 | 38 | 4.11 |
| Fenpyroximate | 111812-58-9 | Insecticide | 0.0328 | 0.0105 | 0.94 | 2.74 |
| Fipronil | 120068-37-3 | Insecticide | 0.0014 | 2.48 | 6.8 | 2.74 |
| Flonicamid | 158062-67-0 | Insecticide | - | - | 11900 | 1.37 |
| Fludioxonil | 131341-86-1 | Fungicide | 2.7 | 2.3 | 2.4 | 50.68 |
| Flufenacet | 142459-58-3 | Herbicide | 309 | 21.3 | 0.2 | 67.12 |
| Fluopicolid | 239110-15-7 | Fungicide | - | 3.6 | 2.9 | 89.04 |
| Fluopyram | 658066-35-4 | Fungicide | - | - | 232 | 100 |
| Flupyradifurone | 951659-40-8 | Insecticide | - | - | - | 4.11 |
| Hexythiazox | 78587-05-0 | Insecticide | 3.6 | 32 | - | 1.37 |
| Imidacloprid | 138261-41-3 | Insecticide | 0.341 | 2110 | 1000 | 21.92 |
| Iprovalicarb | 140923-17-7 | Fungicide | - | - | - | 87.67 |
| Isoproturon | 34123-59-6 | Herbicide | 5.8 | 180 | 1.3 | 84.93 |
| Kresoxim methyl | 143390-89-0 | Fungicide | 1.86 | 1.9 | 6.3 | 45.21 |
| MCPA | 94-74-6 | Herbicide | - | - | 15.2 | 28.77 |
| Mandipropamid | 374726-62-2 | Fungicide | 71 | - | - | 79.45 |
| Mecoprop | 93-65-2 | Herbicide | - | 2400 | 4020 | 35.62 |
| Metalaxyl | 57837-19-1 | Fungicide | 6.4 | 9.6 | 42 | 65.75 |
| Metamitron | 41394-05-2 | Herbicide | 57 | - | 38 | 1.37 |
| Metazachlor | 67129-08-2 | Herbicide | 330 | 85 | 0.23 | 31.51 |
| Metconazole | 125116-23-6 | Fungicide | 42 | 21 | 170 | 10.96 |
| Metobromuron | 3060-89-7 | Herbicide | 441 | 430 | 31 | 5.48 |
| Metolachlor-S | 87392-12-9 | Herbicide | 14 | 12.3 | 1.7 | 95.89 |
| Metrafenone | 220899-03-6 | Fungicide | - | - | 32.7 | 87.67 |
| Myclobutanil | 88671-89-0 | Fungicide | 2.4 | 20 | 266 | 78.08 |
| Napropamide | 15299-99-7 | Herbicide | 54 | 66 | 23.7 | 8.22 |
| Paclobutrazol | 76738-62-0 | Fungicide | 332 | 236 | 0.82 | 8.22 |
| Penconazole | 66246-88-6 | Fungicide | 67.5 | 11.3 | 19 | 83.56 |
| Pencycuron | 66063-05-6 | Fungicide | - | - | - | 4.11 |
| Picoxystrobin | 117428-22-5 | Fungicide | 0.057 | 0.5 | 23 | 1.37 |
| Pirimicarb | 23103-98-2 | Insecticide | 0.17 | 790 | 14000 | 12.33 |
| Propamocarb | 24579-73-5 | Fungicide | 1060 | 968 | 30100 | 64.38 |
| Propyzamide | 23950-58-5 | Herbicide | 39 | - | 2.1 | 35.62 |
| Prosulfocarb | 52888-80-9 | Herbicide | 5.1 | 8.4 | 4.9 | 17.81 |
| Pymetrozine | 123312-89-0 | Insecticide | 617 | - | 2160 | 15.07 |
| Pyraclostrobin | 175013-18-0 | Fungicide | 0.16 | 0.06 | 172 | 10.96 |
| Pyrimethanil | 53112-28-0 | Fungicide | 29 | 105.6 | 120 | 4.11 |
| Spiroxamine | 118134-30-8 | Fungicide | 61 | 71.3 | 0.13 | 82.19 |
| Tebuconazole | 107534-96-3 | Fungicide | 4.6 | 44 | 14.4 | 63.01 |
| Tebufenozide | 112410-23-8 | Insecticide | 8.2 | 30 | 23 | 54.79 |
| Terbuthylazine | 5915-41-3 | Herbicide | 1.67 | 22 | 1.2 | 83.56 |
| Thiacloprid | 111988-49-9 | Insecticide | 0.0912 | 197 | 9670 | 60.27 |
| Thiamethoxam | 153719-23-4 | Insecticide | 0.3409 | - | - | 4.11 |
| Trifloxystrobin | 141517-21-7 | Fungicide | 0.11 | 0.15 | 1.74 | 75.34 |

**Table S 3** Land cover and agricultural land use in a 300 m buffer 3 km upstream of the different sampling sites in the area of Hausen (FR1–FR7) and the Queich catchment (LD1–LD4). Land cover estimates are derived via CORINE CLC 2018 (left side; European Environment Agency 2020b) and agricultural land use was derived with data provided by Blickensdörfer et al. (2021).

| **Land use 300 m buffer 3 km upstream** | | | | | | | | | | | | | |
| --- | --- | --- | --- | --- | --- | --- | --- | --- | --- | --- | --- | --- | --- |
|  | Land cover [%] | | | | | Agricultural land use cover [%] | | | | | | | |
|  | Urban | Industry | Nature | Forest | Agriculture | Vegetables | Spring grains | Winter grains | Fruits | Legume | Grassland | Maize | Other |
| FR1 | 27.18 | - | - | - | 72.82 | 22.20 | 0.57 | 25.62 | 20.07 | - | 3.31 | 18.64 | 9.57 |
| FR2 | 12.47 | - | - | - | 87.53 | 20.53 | 0.42 | 19.24 | 5.98 | 1.08 | 6.05 | 35.61 | 11.08 |
| FR3 | 7.06 | - | - | - | 92.94 | 25.63 | 0.09 | 14.74 | 9.91 | 4.09 | 4.37 | 30.61 | 10.56 |
| FR4 | 1 | - | - | - | 99 | 18.99 | 0.96 | 25.15 | 4.56 | - | 8.12 | 28.54 | 13.67 |
| FR5 | - | - | - | - | 100 | 9.42 | - | 31.15 | 1.07 | 0.71 | 9.80 | 46.34 | 1.51 |
| FR6 | 9.28 | - | - | - | 90.72 | 33.60 | 9.08 | 5.88 | 9.73 | 1.56 | 0.82 | 35.80 | 3.52 |
| FR7 | 19.06 | - | - | - | 80.94 | 26.67 | - | 12.26 | 5.21 | - | 2.83 | 46.91 | 6.12 |
| LD1 | 14.20 | - | - | 65.51 | 20.29 | - | 0.02 | - | 21.58 | - | 75.16 | 0.24 | 3.01 |
| LD2 | 21.36 | - | - | - | 78.64 | - | - | - | 69.26 | - | 21.37 | 1.90 | 7.48 |
| LD3 | 32.34 | 19.65 | 5.22 | - | 42.79 | 12.93 | 4.27 | 9.70 | 12.19 | - | 42.96 | 5.67 | 12.28 |
| LD4 | - | - | - | 35.71 | 64.29 | 17.82 | 0.81 | 2.23 | 7.43 | - | 67.20 | - | 4.50 |

**Table S 4** Land cover and agricultural land use in a 300 m buffer upstream of the different sampling sites in the area of Hausen (FR1–FR7) and the Queich catchment (LD1–LD4). Land cover estimates are derived via CORINE CLC 2018 (left side; European Environment Agency 2020b) and agricultural land use was derived with data provided by Blickensdörfer et al. (2021).

| **Land use 300 m buffer upstream** | | | | | | | | | | | | | |
| --- | --- | --- | --- | --- | --- | --- | --- | --- | --- | --- | --- | --- | --- |
|  | Land cover [%] | | | | | Agricultural land use cover [%] | | | | | | | |
|  | Urban | Industry | Nature | Forest | Agriculture | Vegetables | Spring grains | Winter grains | Fruits | Legume | Grassland | Maize | Other |
| FR1 | 27.18 | - | - | - | 72.82 | 22.20 | 0.57 | 25.62 | 20.07 | - | 3.31 | 18.64 | 9.57 |
| FR2 | 10.31 | - | - | - | 89.69 | 19.43 | 0.47 | 18.42 | 8.70 | 1.40 | 6 | 34.67 | 10.92 |
| FR3 | 7.06 | - | - | - | 92.94 | 25.64 | 0.09 | 14.74 | 9.91 | 4.09 | 4.37 | 30.61 | 10.56 |
| FR4 | 1 | - | - | - | 99 | 18.99 | 0.96 | 25.15 | 4.56 | - | 8.12 | 28.54 | 13.67 |
| FR5 | 4.76 | - | - | 56.47 | 38.77 | 3.51 | 0.59 | 11.26 | 8.77 | 0.34 | 44.99 | 24.26 | 6.19 |
| FR6 | 6.01 | - | 0.45 | 48.26 | 45.28 | 13.31 | 2.07 | 6.64 | 6.29 | 0.31 | 44.79 | 22.28 | 4.31 |
| FR7 | 4.29 | - | 0.60 | 62.16 | 32.95 | 5.34 | 0.54 | 3.45 | 2.33 | 0.01 | 77.61 | 8.12 | 2.59 |
| LD1 | 14.09 | - | - | 69.74 | 16.17 | 0.01 | 0.01 | - | 26.35 | - | 65.21 | 0.11 | 8.24 |
| LD2 | 7.41 | 0.74 | - | 69.45 | 22.40 | - | 0.10 | 0.04 | 49.92 | 0.03 | 40.44 | 1.37 | 8.09 |
| LD3 | 10.56 | 1.48 | 0.26 | 61.25 | 26.45 | 1.33 | 0.33 | 1.32 | 53.35 | 0.02 | 33.66 | 2.03 | 7.95 |
| LD4 | 3.66 | 10.02 | - | 37.97 | 48.35 | 9.97 | 2.04 | 14.90 | 7.70 | 0.70 | 53.52 | 0.65 | 10.53 |
